# Plasma-Driven Surface Functionalization: Enhancing the Corrosion Resistance of Aluminum via Atmospheric-Pressure Treatment

**DOI:** 10.3390/ma19050973

**Published:** 2026-03-03

**Authors:** Carlos A. Cuao Moreu, Reza Jafari, Shamim Roshan

**Affiliations:** Department of Applied Sciences, Université du Québec à Chicoutimi (UQAC), 555 Boul. de L’université, Chicoutimi, QC G7H 2B1, Canada; cacmoreu@etu.uqac.ca (C.A.C.M.); sroshan@etu.uqac.ca (S.R.)

**Keywords:** atmospheric pressure plasma, aluminum, plasma treatment, aluminum oxide, functionalization, corrosion resistance

## Abstract

Aluminum and its alloys are employed in different industries due to their versatile properties (low density, resistance to corrosion, thermal conductivity). However, the corrosion properties of these alloys can be enhanced through protection methods. In this paper, the effect of surface treatment through atmospheric pressure plasma on the corrosion properties of a 6061 aluminum alloy was evaluated. The corrosion resistance was evaluated using electrochemical impedance spectroscopy. The jet-substrate distance and number of passes were selected as the plasma process parameters. The plasma treatment allowed the formation of a passive layer that exhibits higher corrosion resistance than the untreated substrate, corresponding to an increase of nearly three orders of magnitude in polarization resistance. Further, the passive layer’s morphological characteristics and chemical composition were analyzed through scanning electron microscopy with energy dispersive spectroscopy, optical profilometry, and X-ray photoelectron spectroscopy, where an increase of around 40% in oxygen content supports the presence of an oxygen-rich layer on the surface.

## 1. Introduction

The most common corrosion process in nature is atmospheric corrosion, associated with millionaire financial losses due to the degradation of metallic structures employed in different industries (construction, oil and gas, automotive, aerospace, and marine) [1]. Among the different metallic materials used to fabricate industrial components, aluminum and its alloys are widely employed, considering their broad range of properties like low density, a wide range of strength values (depending on the alloying elements), high resistance to corrosion under different service conditions, excellent conduction of heat and electricity, non-ferromagnetism, and ease of recycling and fabrication [2].

Employed as a structural material in transport, building, aircraft, and aerospace [1,3,4], 6061 aluminum (Al) alloy is one of the most used Al–Mg–Si series aluminum alloys. However, during exposure to different atmospheric environments, the surface of 6061 Al alloy can be affected by distinct corrosion forms like pitting and intergranular corrosion [1,5]. The ability of aluminum to resist corrosion in oxidizing environments, such as air or water, is due to its natural surface oxide layer (Al_2_O_3_), which is only a few nanometers thick. This layer inhibits oxidation. However, when aluminum is subjected to high concentrations of acid or alkaline solutions, this protective oxide layer can dissolve, leaving the surface susceptible to corrosion. Additionally, localized attacks from aggressive anions, particularly halides like chloride ions, can compromise this stable layer in solutions with pH levels ranging from 4 to 9 [6]. As a result, various strategies have been employed to enhance the corrosion resistance of aluminum alloys across different conditions. Organic or inorganic barrier coatings are typically used to mitigate the corrosion rates of aluminum alloys; examples of these coatings include chromium conversion coatings, electrodeposited ceramic coatings [7], and polymeric coatings [8,9]. However, the use of coatings implies some disadvantages like distortion, cracking, delamination, and harmful effects from inadequate atmospheric protection (inclusions and contaminants) [10].

On the other hand, there are conversion protection techniques, which involve converting the metal surface into a protective layer against corrosion [11]. Different protection mechanisms against corrosion have been employed on 6061 Al alloys. Paksoy et al. [12] carried out a micro-arc oxidation (MAO) treatment on a 6061 Al alloy substrate to create aluminum oxide layers in the form of ɑ-alumina and ϒ-alumina as an additional corrosion barrier, since the natural oxide layer is not sufficiently protective against chloride-containing media. The results of the corrosion immersion test in a 3.5% mass fraction of NaCl solution revealed that the weight gained by the MAO samples was lower than the untreated samples, which was related to the sealed effect of the MAO treatment that controlled the deposition of corrosion products. On the other hand, Charitha et al. [13] studied the response to corrosion of 6061 Al alloy when it is immersed in a 37% HCl solution containing dextran as a corrosion inhibitor. It was found that the negatively charged metal surface attracts the dextran molecules from the corrosive media and forms a protective physical barrier, which reduces the electrochemical corrosion process according to Electrochemical Impedance Spectroscopy (EIS) tests.

During the last few years, plasma technology has emerged as a promising alternative for improving the corrosion resistance of metallic surfaces [14,15,16]. The energy produced during plasma can be employed to activate the surface for further coatings deposition [16], create thin films (deposition) [17], form ultra-thin polymer-like layers (polymerization) [18,19], etc. In the case of 6061 Al alloy, research on plasma technology for corrosion protection has been focused on plasma electrolytic oxidation (PEO), where a high voltage is applied through an electrolyte, generating plasma discharges on the substrate surface [20]. Different authors [21,22,23] have found that the anticorrosive properties of the PEO coatings are based on the reduction of microcracks and pores more than the increase of the coating thickness. The coating characteristics can be manipulated by optimizing the processing parameters (current density, frequency, voltage, time, electrolyte composition, etc.). Even so, the PEO process requires high energy consumption [24]; the high power and current frequency required are the principal disadvantages using the PEO at a commercial upscaling [25]. The intense micro-discharge activity during PEO can also be associated with residual stress that influences coating delamination or poor adhesion under cyclic loading [26]. Additionally, there are still some limitations in terms of reproducibility related to the non-homogeneous coating growth for complex geometries [27].

Nevertheless, a plasma surface treatment where the reactive species (electrons, radicals, ions, and neutral particles) obtained through plasma react with the surface and form a thin film has yet to be explored for aluminum alloys. In addition, this treatment has exhibited promising anticorrosive properties on steels [28,29]. One of this technique’s main advantages is the opportunity to employ an atmospheric pressure system, which reduces the capital costs of equipment and eliminates constraints imposed by vacuum compatibility [14]. Also, the atmospheric pressure plasma treatment allows treatment of larger areas compared to other systems [30]. One study carried out on the impact of the atmospheric pressure plasma treatment on the corrosion properties of the formed oxide layers on the surface of AA1070 and AA2024 alloys evidenced a lower corrosion current density [14], showing a strong potential for developing anticorrosion aluminum surfaces.

This paper evaluated the effect of atmospheric plasma treatment on the corrosion properties of a 6061 aluminum alloy. Thin film oxides were obtained on the aluminum surfaces, varying the distance jet-substrate and the number of passes. The corrosion resistance was evaluated through EIS, and the morphological and chemical characterization was conducted using Scanning Electron Microscopy (SEM) with Energy Dispersive Spectroscopy (EDS), 3D Profilometry, and X-ray Photoelectron Spectroscopy (XPS).

## 2. Experimental Details

The substrate was 6061 T6 aluminum alloy (AA6061) (1%Mg, 0.6%Si). The samples were cut into square specimens with an area of 25 cm^2^ and a thickness of 1 mm. The surface was ultrasonically cleaned for 5 min in acetone before the plasma treatment. A commercial OpenAir AS400 atmospheric pressure plasma jet (APPJ) manufactured by PlasmaTreat^®^ GmbH (Steinhagen, North Rhine-Westphalia, Germany) with a PFW10 nozzle was used. The process was conducted at room temperature, exposed to ambient air. The plasma parameters are listed in Table 1. These parameters were fixed based on some preliminary trials, where this combination allowed creation of a uniform plasma oxidized layer on the aluminum samples.

The effect of two remaining factors—distance jet-substrate and number of passes—on the anticorrosion properties of the plasma treated surfaces was assessed based on previous work related to optimizing atmospheric plasma parameters to increase the functionality of surfaces [31,32,33]. Two values were selected: 3 and 4 mm for the distance jet-substrate, and 4–6 for the number of passes. Within this parameter range, sufficient plasma density and reactive species flux are produced to promote surface activation. Shorter distances or excessive passes may cause excessive energy transfer or structural degradation, whereas larger distances reduce plasma density to modify the surface. The nomenclature of the employed samples is exhibited in Table 2.

Prior to any tests, the surface of the plasma-treated samples was ultrasonically cleaned for 5 min in acetone. The corrosion resistance was measured at room temperature employing a Biologic SP-300 potentiostat (Bio-Logic Science Instruments, Seyssinet-Pariset, France) under the control of EC-Lab software (version v11.50). Three electrodes were used for the electrochemical measurements, the working electrode being the sample, the reference being an Ag/AgCl electrode and the counter electrode being a graphite electrode. The exposed working area was 1 cm^2^, and a 0.05 M NaCl solution was used as electrolyte. Before each experiment, the samples were immersed in the electrolyte solution for 24 h. EIS tests were performed by applying an alternating current signal with an amplitude of ±10 mV in a frequency range from 10 kHz to 0.1 Hz.

To better understand the corrosion phenomena, the roughness of the surface was obtained by the average of at least three measurements on different surface areas using an optical profilometer Profilm3D Filmetrics^®^ (Filmetrics, Inc., San Diego, CA, USA). Additionally, the surface morphology and chemical composition of the modified aluminum surfaces was analyzed by SEM-EDS JEOL JSM 6480 LV (JEOL Ltd., Tokyo, Japan). The chemical bonds were analyzed by X-ray photoelectron spectroscopy (XPS) using a Physical Electronics PHI 5600-ci instrument (Chanhassen, MN, USA). A standard aluminum X-ray source (1486.6 eV) was used to record both survey and high-resolution spectra without charge compensation. Detection was carried out at an angle of 45° concerning the surface normal, and the analyzed area was 0.5 mm^2^. Three measurements for each sample were taken to confirm the homogeneity of the chemical composition. The curve fitting procedures were done employing a least-square Gaussian–Lorentzian peak fitting procedure, after Shirley background subtraction. The C1s peaks were set at 285 eV (C-C and C-H) as reference.

## 3. Results and Discussion

**Figure 1:**
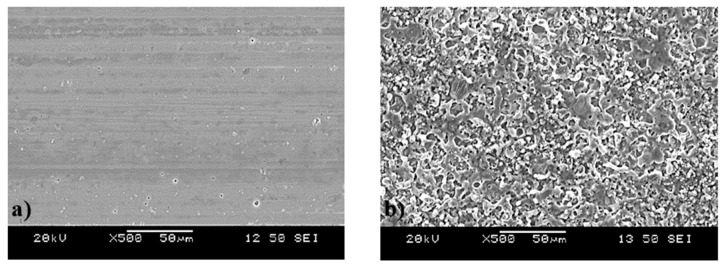
Superficial SEM micrographs of the 6061 aluminum samples: (**a**) untreated and (**b**) plasma treated.

SEM images of the superficial area of the untreated and atmospheric plasma-treated samples are shown in Figure 1. The surface of the untreated sample exhibited the aluminum surface without any modification; instead, a porous microstructure was observed on the plasma-treated sample. The uniform micro-porous structure was observed over the whole surface treated with plasma. This formation of the porous structure could be related to the formation of volatile products after the impact of the ionized gas from plasma with the elements on the surface [34]. Also, the pressure at which the ionized gas impacted the porous structure could have influenced its morphology [35].

**Figure 2:**
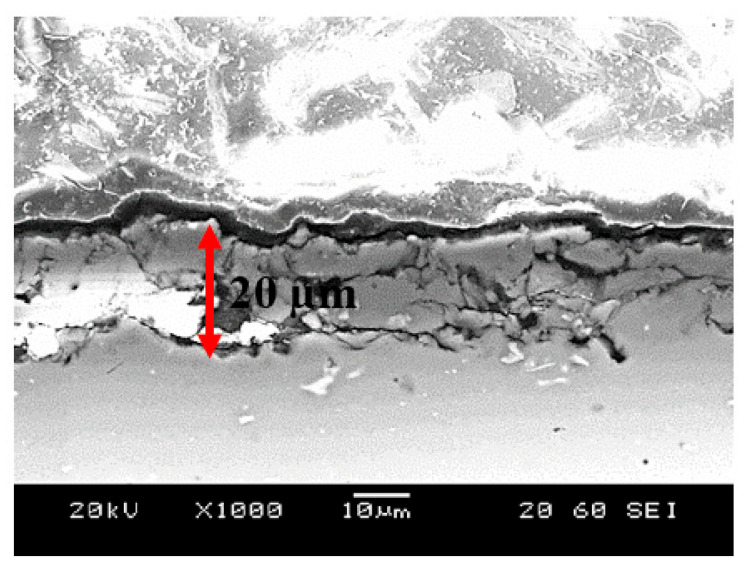
Cross-section SEM micrograph of the 6061 aluminum treated with plasma (S3 sample).

Further, the cross-sectional SEM image of the plasma-treated samples is exhibited in Figure 2. Cracks could be observed on the top of the surface. This zone where the cracks appeared may correspond to the porous structure. The formation of cracks could be associated with the rapid cooling of the aluminum surface in air after the plasma treatment, which caused an embrittlement of the surface. It has also been reported [36] that the thin layers obtained through plasma on the metallic surfaces exhibit a higher hardness than the bulk material because of the formation of new compounds.

**Figure 3:**
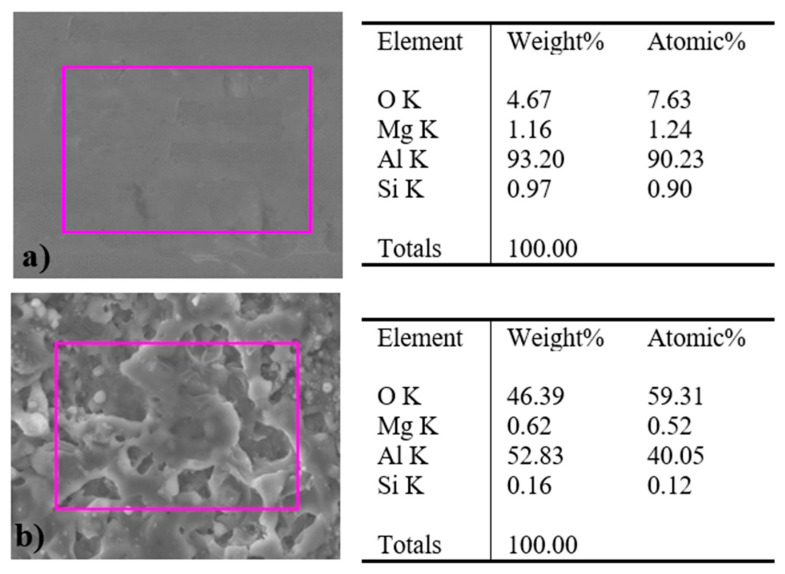
EDS elemental analysis (rectangle frames) over the surface of the 6061 aluminum: (**a**) untreated and (**b**) plasma treated.

**Figure 4:**
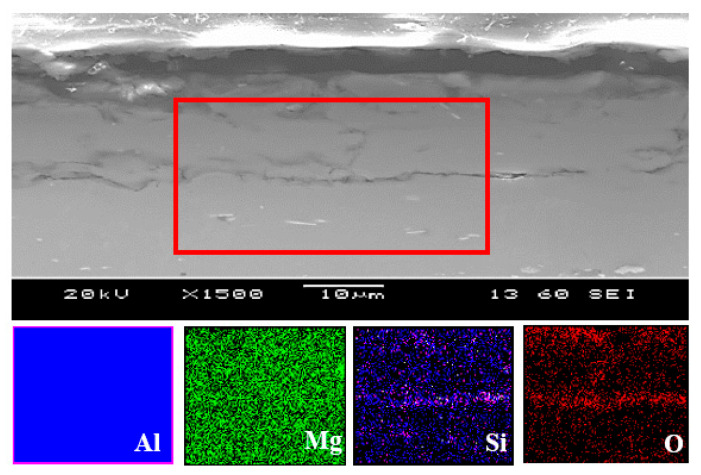
EDS elemental mapping over the cross-section (rectangle frame) of the 6061 aluminum treated with plasma.

Additionally, the elemental chemical changes of the untreated and plasma treated were obtained through SEM-EDS, and it is exhibited in Figure 3. An increase in the percentage of oxygen after the plasma treatment was notorious. It could be related to the oxidation on the surface after the reaction between the elements on the surface and the species from plasma [34], since the process was carried out in the air. In the EDS mapping (Figure 4) of the plasma-treated sample, the chemical distribution of Al, Mg, and Si (blue, green, and violet, respectively) is uniform since they are the elements present in the 6061 aluminum alloy. In the case of O (red), a different distribution was observed. A higher concentration of O was detected in the upper part, where the plasma layer was located, as shown in Figure 2. This could be related to the oxidation of the surface after plasma functionalization mentioned previously.

**Figure 5:**
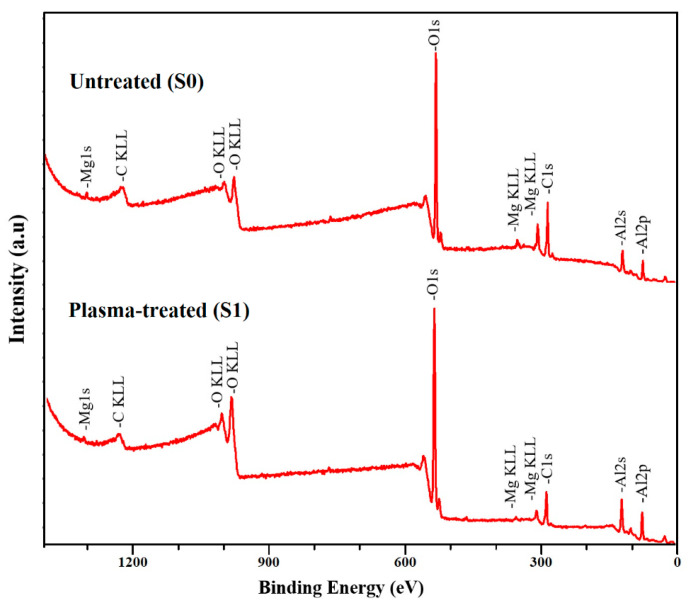
XPS survey spectra of untreated and plasma-treated samples.

**Figure 6:**
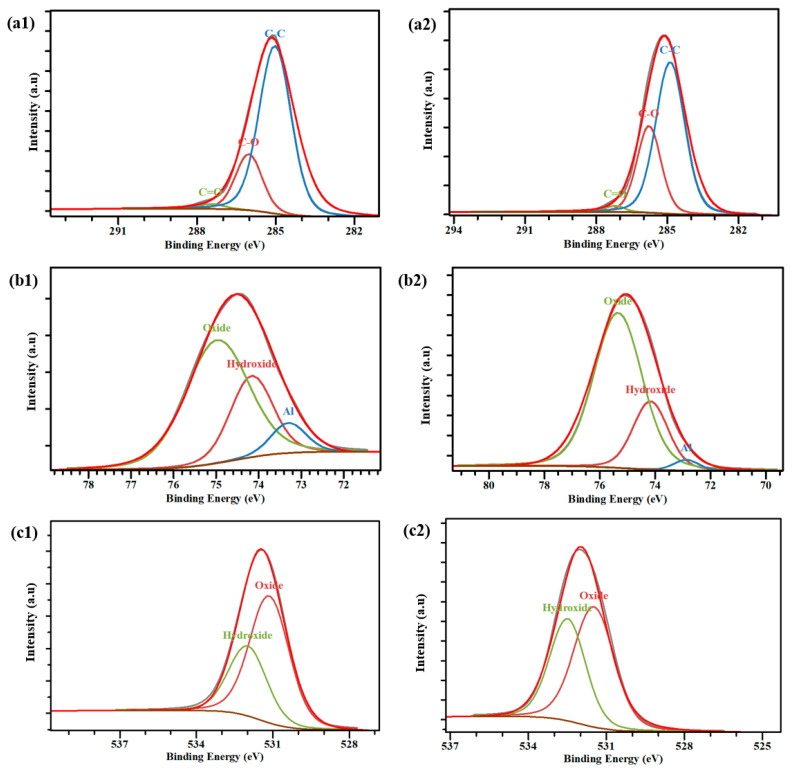
Typical view of the C1s peak measured at high-resolution on the (**a1**) untreated (**a2**) plasma treated samples, Al2p aluminum samples peak measured at high-resolution on the (**b1**) untreated (**b2**) and O1s peak measured at high-resolution on the (**c1**) untreated (**c2**) plasma treated samples.

XPS analysis was conducted on the untreated aluminum surface and the plasma-treated samples. Due to the presence of a native oxide layer, aluminum surfaces are prone to adsorbing contaminants, particularly from the surrounding environment. Therefore, the carbon-related signals observed in both the bare and plasma-treated aluminum surfaces were thoroughly examined. Figure 5 displays a representative XPS survey spectrum of both untreated and plasma-treated samples. The dominant peaks consistently observed across all spectra of the aluminum specimens correspond to O1s, Al2p, Al2s, and C1s signals. Table 3 summarizes the surface composition of the samples before and after plasma treatment under optimal conditions. The distinct carbon signal, particularly the hydrocarbon peak at 285.0 eV in the C 1s spectrum, was utilized as an internal standard for calibrating the binding energy scale. Following this calibration, the curve fitting of the high-resolution C 1s peaks was conducted employing a hybrid Gaussian–Lorentzian function. Figure 6 illustrates the high-resolution C 1s spectra derived from the XPS measurements of both untreated and plasma-treated 6061 aluminum alloy, typically involving a complex deconvolution.

Elevated oxidation states of carbon for the untreated sample, observed at 285.8 eV and 287.4, are indicative of C-O and C=O functional groups respectively and for the plasma treated sample they appeared at 285.7 and 287.3 eV.

For the plasma-treated AA6061, there was a notable increase in intensity observed across both low and high oxidation states of carbon. Moreover, the carbon content on the surface decreased from 76.2 at.% to 60.1 at.%. The observed decrease in carbon content shows the modification effect of plasma treatment on the aluminum surface. In addition, the ratio of O/C is increased from 0.64 to 2.5 after plasma treatment, probably due to the oxide and hydroxide appearing on the aluminum surface.

The oxidation facilitated by plasma radicals, coupled with the impact of gas flow, contributed to surface cleaning. Nevertheless, substantial carbon residues in low oxidation states remained on the surface of the plasma-treated AA6061. The presence of residual carbon on this sample can be attributed to the adsorption of carbonaceous species such as dust, CO_2_, hydrocarbons which are present in air, onto the native aluminum oxide layers.

Figure 6(b1,b2) illustrates the influence of plasma treatment on the aluminum 2p spectral peak for both the untreated and plasma-treated aluminum AA2024 samples.

As depicted in Figure 6(b1,b2), the aluminum spectrum is deconvoluted into three main peaks: the peak at a binding energy of 74 and 74.1 eV, which corresponds to the elemental aluminum hydroxide, and the predominant peak at 75.6 eV and 75.3 eV are related to the aluminum oxides, and the minor peaks appearing at 72.8 and 73.2 correspond to the elemental aluminum component (Al) [37].

Based on the deconvoluted peaks of aluminum, the separation of aluminum peaks between the untreated and plasma-treated aluminum AA6061 showed no significant variance, indicating that the chemical structure of the oxide layers generated by plasma treatment closely resembles that of the native oxide layers [38,39].

The relative area ratio of the elemental aluminum component (Al) peaks and Al oxide peaks in Al spectra are strongly associated with the thickness of the aluminum oxide layer. When the thickness of the hydrocarbon overlayer is high on the as-received aluminum surface, an accurate quantitative evaluation of the native aluminum oxide thickness based on the Al 2p signal was not achievable. So, in this work, the intensity of the oxidized aluminum component was utilized as a qualitative indicator for comparative assessment of oxide layer thickness among different samples [37,40]. Based on Figure 6(b1,b2), the intensity of the aluminum oxide peak on the plasma treated surface was 73.7 at. % and for the untreated sample, 61.9 at. %. This increase is likely responsible for the elevated polar component of the surface energy and aligns well with findings reported in previous studies [41].

References [39,42] indicate that exposure to atmospheric conditions leads to the hydration of the surface aluminum oxide layers on aluminum alloys. The increase in the hydration state is more obvious in the O 1s XPS spectra. Figure 6(c1,c2) illustrated various O 1s XPS spectra of untreated and treated AA6061 samples with atmospheric plasma, respectively. The results show a significant increase in intensity of O 1s peak in the plasma-treated AA6061 sample, which is attributed to the oxidation effects of radicals produced by the plasma process. The peaks obtained from deconvolution of the O 1s spectra can be attributed to a thin layer of aluminum hydroxide and oxide species present on the upper layer of the aluminum substrates [40,43]. In the untreated sample, the peak at 531.3 eV is indicative of aluminum oxide and also an oxide form of carbon, while the peak at 532.8 eV corresponds to hydroxide [38]. The mentioned peaks for the treated sample were observed at 531.4 eV and 532.5 eV. Based on the O1s peak, the concentration of hydrated components significantly increased from 34.8 at. % to 44.9 at. % when the surface is treated with atmosphere plasma, and the reason could be the adsorption of hydroxyl radicals during the plasma process.

**Figure 7:**
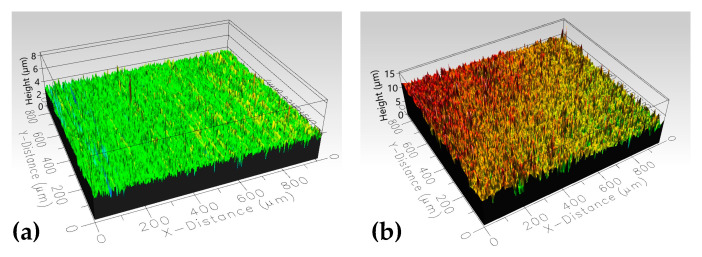
Images and Sa roughness values obtained by profilometry of the: (**a**) untreated sample and (**b**) plasma treated sample.

The morphological changes on the aluminum surface were analyzed through profilometry, and the results are exhibited in Figure 7. The surface roughness of the plasma-treated samples increased according to the measure of the arithmetic mean height (Sa) in Table 4. It could be associated with the formation of the micro-porous structure, which could have increased the roughness. Additionally, chemically reactive species are introduced to the surface through plasma, which can modify the morphology of the surface depending on the surface properties, temperature of the material, fluxes of reactive species onto the treated material, and treatment time [44]. The roughness values of the plasma-treated samples were consistently similar. However, sample S2 exhibited the highest roughness value, which could be associated with the degree of exposition of the surface with the plasma, since the plasma conditions applied on this sample were the most severe (3mm distance jet-substrate and six passes).

**Figure 8:**
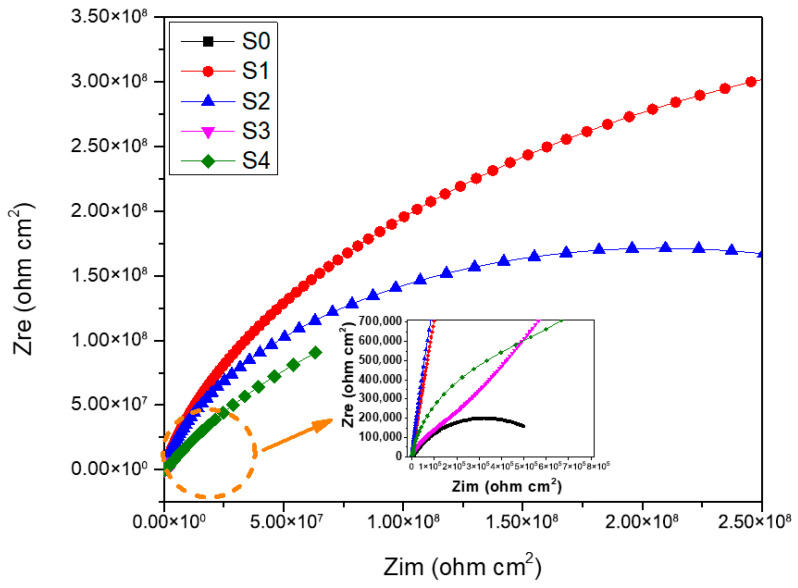
Nyquist diagrams for the untread and plasma-treated aluminum samples.

**Figure 9:**
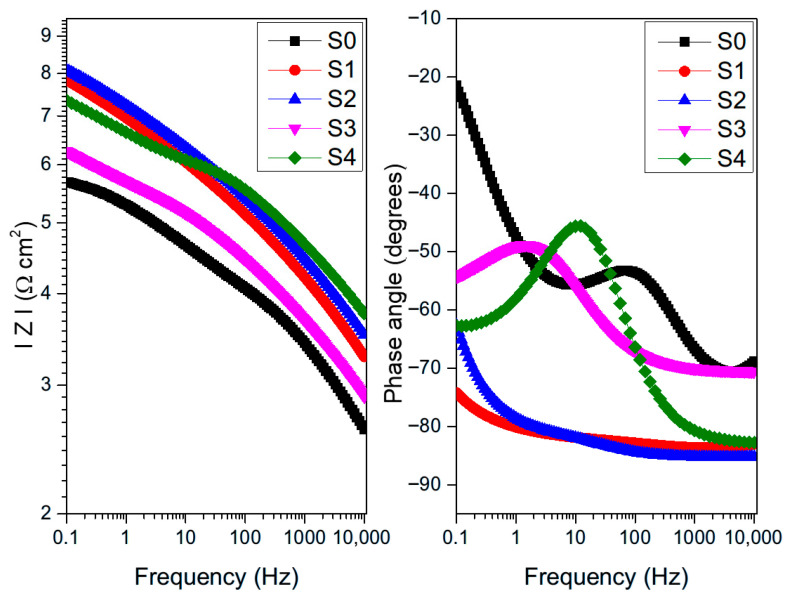
Bode plots for the untreated and plasma-treated aluminum samples.

**Figure 10:**
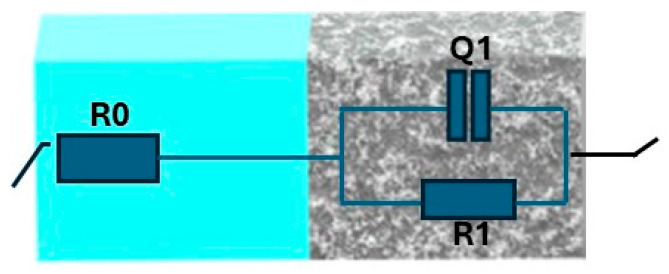
The equivalent circuit is used for the quantitative evaluation of EIS.

The electrochemical behavior of the untreated sample and the samples treated with plasma process is assessed by EIS measurements in 0.05 M NaCl solution. Figure 8 and Figure 9 show the Nyquist and Bode diagrams examining the effect of the plasma treatment parameters on the anti-corrosion performance of the 6061-T6 aluminum alloy, respectively. The semicircles in Nyquist diagrams are the electrochemical response of the surface during the impedance tests. The diameter of the capacitive loop is indicative of the corrosion resistance of the samples; an increase in the semicircular arc suggests improved surface protection efficiency. As shown in Figure 8, plasma treatment could significantly enhance the anti-corrosion performance of the aluminum substrate. Figure 10 is used to simulate the impedance data. The data obtained from the electrical equivalent circuit (EEC) are listed in Table 5. An oxide layer was obtained on the aluminum surface during the plasma functionalization. Thus, the selected circuit describes the electrochemical behavior of passive metallic substrates, where an interaction occurs between the native oxide film and the underlying space charge layer during the impedance response [45]. For the sample S0, the equivalent circuit is associated with the native aluminum passive film, whose behavior is similar to that of other passive metals described in the literature [46,47]. In this kind of configuration, the high-frequency time constant corresponds to the barrier properties of the passive film, and the intermediate-frequency response is associated with the ionic transport and its interaction with the defects within the passive film. In the employed equivalent circuit, R_0_ is the resistance of the solution; R_1_ is the polarization resistance related to the material’s corrosion resistance. Q_1_ represents a constant phase element associated with the non-ideal capacitive behavior of the substrate’s surface in contact with the electrolyte [48]. For the S0 sample, Q_1_ is associated to the native aluminum oxide, whereas for the plasma-treated samples it is related to the plasma-grown oxide. In both cases, this constant indicates the dielectric response of the passive film and also its ion transport characteristics. Since the oxide layers provided by plasma treatments are usually non-homogenous in their thickness and composition, they cannot act as an ideal capacitor, so the constant phase element is used instead of an ideal capacitor [29]. The impedance representation of a constant phase element is given by [14]:

$$Z\left(CPE\right)={1/[Q\left(j\omega \right)}^{n}]$$

where *ω* is the angular frequency in rad s^−1^, *Q* is a constant phase element, and *n* is an empirical exponent that can be changed between 0 for a perfect resistor and 1 for a perfect capacitor.

The term “capacitance” refers to the behavior of a metal coating (in this case: native oxide for the sample S0, and plasma layer for the other samples) immersed in an electrolyte.

The distinct enlargement of the capacitive semicircles in the treated samples could be related to a significant reduction in charge transfer kinetics, due to the formation of a thicker and more compact oxide barrier (Figure 2) induced by plasma exposure [14]. According to the results of sample S0, the native oxide layer remains permeable to the corrosive species, which leads to a relatively low polarization resistance compared to the other samples. For the plasma-treated samples, the oxide layer generated by plasma does not develop independently from the native oxide layer, resulting in overlapping loops observed in the Nyquist plots. The overlapping semicircles could be an indication that the plasma-grown oxides does not replace but instead reinforces the native oxide, producing a kind of multilayered configuration where the plasma layer acts as the primary barrier for chloride ingress [49]. The corrosion properties of the oxides formed on the surface of aluminum alloys after atmospheric pressure plasma jet treatment have been previously studied by K. Brunelli et al. [14], where an AA1070 aluminum alloy was employed, and the impedance reached values around 5.0 × 10^4^ Ω cm^2^. However, in the current research, the impedance increased until values around 3.0 × 10^8^ Ω cm^2^, which indicates the potential of the atmospheric plasma to improve the corrosion protection of metallic surfaces. The impedance of the plasma-treated samples (S1 and S2) increased by nearly four orders of magnitude compared to literature [14], suggesting enhanced plasma density and energy transfer at 3 mm distance.

The higher capacitive semicircles of samples S1 and S2 compared to samples S3 and S4 could be related to the shorter distance jet substrate. A dense plasma layer could be formed on the samples S1 and S2 with 3 mm of distance jet-substrate. The superior performance of S1 relative to S2 could be attributed to a trade-off between oxide densification and excessive heat accumulation during additional passes. In the case of sample S2, the excessive thermal exposure could have induced local stresses, reducing the overall protective effect [14,50]. Nevertheless, sample S4 showed a higher capacitive semicircle compared to sample S3, being the number of passes greater for sample S4, but in this case the shorter distance jet-substrate could have reduced the local stress.

On the other hand, bode plots for both the untreated and treated aluminum samples are shown in Figure 9. The impedance measured in the low-frequency region (0.1 Hz) of Bode module reflects the overall impedance and corrosion resistance of the samples, and is approximately equivalent to the polarization resistance [50]. According to the relationship log |Z|–log frequency, the plasma-treated samples exhibited a higher impedance module compared to the untreated samples, which is consistent with the capacitive semicircles with increased diameters of the Nyquist diagrams. The samples S3 and S4 exhibited the lowest impedance module of the plasma-treated samples. It may be related to the higher distance jet-substrate, the critical variable that controls the formation of the plasma layer with anticorrosive properties.

From the relationship phase angle–log frequency of the bode plots, the S0 sample exhibited a jump from 70 to 50 degrees in the range from 10,000 Hz to 100 Hz. This increase, which is associated with a time constant in an intermediate-frequency region (10–1000) Hz, may be related to a decrease in the capacitive properties of the native oxide on the aluminum surface. At high frequencies (10 kHz), the response is dominated by the properties of the native oxide on the aluminum surface acting as a capacitor. The mid-frequency peak (100 Hz) indicates a secondary relaxation process, possibly related to defects or porosity in the oxide film, or interactions at the oxide/metal interface. The wider phase angle dispersion in sample S0 could be inferred as a more defective and porous native oxide, where the heterogeneous distribution of the oxide thickness and the presence of microstructural discontinuities could have influenced multiple relaxation processes [51].

In the case of samples S1 and S2, the formation of a plateau that kept an almost constant phase angle value was observed. This stable phase angle behavior could indicate the capacitive response of the aluminum oxide formed after the plasma treatment. The samples S3 and S4 exhibited one peak at 1 and 10 Hz, respectively. In the low-frequency region (≤10 Hz), the formation of time constants may be associated with the diffusion of species, in this case, probably the diffusion of species through the plasma layer. Therefore, the passive layers formed on samples S3 and S4 were not as dense as the passive layers formed on the samples S1 and S2 since there was diffusion of species through them. It may be related to the higher distance jet-substrate mentioned above.

Table 5 summarizes the calculated parameters obtained for fitting the EIS data. The solution resistance (R_0_) was almost constant for all samples, which is related to the stability of the electrolyte conditions.

The polarization resistance (R_1_) is higher for the plasma-treated samples than for the untreated samples, indicating that the creation of oxides after the plasma functionalization enhances corrosion resistance.

Additionally, the constant phase element (Q_1_) values are lower for most plasma-treated samples in comparison with the untreated sample. Its magnitude is influenced by the dielectric properties of the oxide and structural heterogeneity [38,39]. The marked reduction in Q_1_ for samples S1 and S2 could be related to the formation of a thicker and more compact oxide layer during plasma treatment. Moreover, the value of the exponent n_1_ close to 0.9 for S1 and S2 compared to 0.67 for S0 indicates a less defective plasma-layer [52]. However, the Q_1_ was similar between the S4 and S0 samples, which could explain the lower anticorrosive properties of this plasma condition. Additionally, the relatively higher Q_1_ values and lower n_1_ exponents observed for samples S0, S3, and S4, together with their reduced R_1_ values, in comparison with the samples S1 and S2 may indicate increases surface heterogeneity and the possible effect of localized corrosion. Lower n_1_ values are commonly associated with defective or partially degraded passive films, and in this case, the constant phase element may reflect both the dielectric behavior of the oxide film and also the contribution of the electrical double layer in locally corroded areas [50]. For sample S0, this behavior corresponds to the native oxide layer, whereas for samples S3 and S4 it is associated with the lower protective performance of the plasma-formed oxides under these processing parameters.

Despite XPS results showing that the plasma-formed oxides have a chemical structure similar to the native aluminum oxide, the improvement in the corrosion resistance could be mainly attributed to the physical modifications of the oxide layer. The increased intensity of the Al components after plasma treatment suggests a thicker oxide layer, while the reduction in Q_1_ values and increase in R_1_ could be related to enhanced compactness and reduced electrolyte permeability. Even though the hydrated components of oxide increased, it could have played a secondary role. The dominant mechanism was then associated with the formation of a denser oxide barrier that decreases chloride diffusion and charge transfer processes [14,53].

Although porosity was observed on the plasma-treated surfaces, it seems to be mainly superficial. Unlike conventional PEO coatings [54,55], where interconnected pores induce electrolyte penetration, the oxide layer produced by the present plasma system generates an oxide layer that acts as a compact barrier (Q_1_ and R_1_ behavior).

## 4. Conclusions

Based on the present study of the plasma functionalization process on the corrosion behavior of a 6061 aluminum alloy in a 0.05 M NaCl solution, the main findings can be summarized as follows:

Atmospheric pressure plasma treatment induces oxidation of the aluminum surface, which increases the percentage of oxygen from 33 at.% (S0) to 52.3 at.% (S1), which corresponds to an increase of around 40% related to the formation of an oxygen-rich oxide layer.

A porous structure was produced by the plasma treatment, which increased the surface roughness (Sa) from 0.31 µm (S0) up to 1.16 µm (S2), depending on processing parameters.

The plasma treatment increased the corrosion resistance of the 6061 aluminum surface. Moreover, based on the EIS results, the distance jet-substrate played a key role in producing a dense plasma layer that increased its impermeability and improved the corrosion resistance. The optimal condition (3 mm distance) produced a polarization resistance increase of nearly three orders of magnitude compared to the untreated sample. However, the effect of the number of passes on the corrosion resistance remained unclear.

The improvement in the corrosion resistance was primarily attributed to the formation of a denser and less defective oxide barrier, instead of changes in the chemical structure. However, the oxide layers formed under the less favorable plasma conditions exhibited greater surface heterogeneity and electrochemical features consistent with possible localized corrosion, according to the impedance parameters.

## Figures and Tables

**Table 1:** Plasma parameters employed during surface treatment.

Parameters	Values
Velocity jet slide	1 m/min
Percentage reference voltage (340 V)	100%
Plasma frequency	21 kHz
Cycle time	100%
Gas flow rate	3000 L/h

**Table 2:** Description of the employed samples.

Sample Name	Employed Variables	
	Distance Jet-Substrate [mm]	Number of Passes
S0	Untreated sample	
S1	3	4
S2	3	6
S3	4	4
S4	4	6

**Table 3:** Elemental composition (at.%) of the untreated (S0) and plasma-treated (S1) aluminum AA2024-T3.

Samples Code	O	Al	C	O/C
S0	33.1	15.3	51.1	0.64
S1	52.3	26.3	20.8	2.5

**Table 4:** Arithmetic mean height (Sa) obtained by profilometry.

Sample	Sa (Arithmetic Mean Height) [µm]
S0	0.3121
S1	0.6697
S2	1.1697
S3	0.7472
S4	0.7457

**Table 5:** Values of the equivalent circuit in 0.05 M NaCl.

Sample	R_0_ (Ω cm^2^)	Q_1_ (S s^n^ cm^−2^)	n_1_	R_1_ (Ω cm^2^)
**S0**	45.24	1.20 × 10^−6^	0.67	6.69 × 10^5^
**S1**	44.18	2.13 × 10^−8^	0.90	6.54 × 10^8^
**S2**	43.19	10.7 × 10^−9^	0.92	3.8 × 10^8^
**S3**	45.15	5.66 × 10^−7^	0.67	3.47 × 10^6^
**S4**	49.18	1.02 × 10^−6^	0.72	1.93 × 10^6^

## Data Availability

The original contributions presented in this study are included in the article. Further inquiries can be directed to the corresponding author.
